# Changes of macrophage and CD4^+^ T cell in inflammatory response in type 1 diabetic mice

**DOI:** 10.1038/s41598-022-19031-9

**Published:** 2022-09-02

**Authors:** Chenhao Li, Qingyuan Gao, Hao Jiang, Chengrun Liu, Yujun Du, Lisha Li

**Affiliations:** 1 Department of Nephrology, The First Hospital of Jilin University, Changchun, 130021 Jilin Province China; 2 The Key Laboratory of Pathobiology, Ministry of Education, College of Basic Medical Sciences, Jilin University, Changchun, 130021 Jilin Province China

**Keywords:** Immunology, Diseases, Endocrinology

## Abstract

Immune cells play an important role in the development of inflammation in type 1 diabetes mellitus, so we want to explore the changes of CD4^+^ T cells and macrophages in vivo, which can provide an experimental basis for immunotherapy based on CD4^+^ T cells and macrophages. The intraperitoneal injection of streptozocin was used to induce a type 1 diabetes mellitus mouse model; the blood glucose, body weight, and the expression of inflammatory factors in the kidney were measured. Immunohistochemistry was applied to determine and analyze the infiltration of CD4^+^ T cells and macrophages in the spleen, pancreas, and kidney. The subtypes of macrophages in the kidney and CD4^+^ T cells in the spleen were analyzed by flow cytometry. Our study suggests that CD4^+^ T cells and macrophages increase, while the inflammatory immune response system is activated in the development of T1DM. CD4^+^ T cells positively correlated with macrophages in the pancreas and kidney of T1DM. CD4^+^ T cells turn to pro-inflammatory subtypes in the spleen of T1DM, while macrophages turn to pro-inflammatory subtypes in the kidney of T1DM. Therefore, regulation of CD4^+^ T cells and macrophages may be a potential target for T1DM and kidney complications.

## Introduction

Type 1 diabetes mellitus (T1DM) is an autoimmune disease caused by immune-mediated destruction of pancreatic islet cells^1^. The typical pathological features of pancreatic islets are lymphocyte infiltration, which leads to the destruction of islet β cells, responsible for insulin production and secretion^2^. T1DM is characterized by insulin deficiency.

CD4^+^ T cells play an important role in the pathogenesis of T1DM^3^. CD4^+^ is a surface marker of T helper (Th) cells. CD4^+^ T cells can differentiate into anti-inflammatory subtypes, such as Type 2 helper (Th2) cells, and pro-inflammatory subtypes, such as Type 1 helper (Th1) cells and Type 17 helper (Th17) cells; T regulatory cells (Tregs) are one necessary CD4^+^ T subtypes in preventing autoimmunity. CD4^+^ can also assist other lymphocytes to perform the immune function and modulate the immune response. In T1DM mice, the proportion of pro-inflammatory cells Th1 cells and Th17 cells increased and induced persistent pancreatic inflammation. At the same time, IFN- γ And IL-1 secreted by Th1 cells damage islets β cellular function^4^. Insulitis was observed in mice injected with CD4^+^ T-cell from diabetic donors and CD4^+^ T-cell can also cause a targeted, destructive infiltration of pancreatic β-cells^5^. CD4^+^ T cells initiate autoimmune attack of pancreatic islet β- cells^6^. Therefore, macrophages and CD4^+^ T cells play an important role in the inflammatory process of T1DM.

Macrophages are one of the most important innate immune cells which exist widely on the surface of tissue mucosa. Macrophages can activate the acquired immune response, release cytokines, participate in inflammatory reactions and maintain immune homeostasis. Macrophages exhibit significant plasticity in different microenvironments. Macrophages are classified into classically activated macrophages (M1) and alternatively activated macrophages (M2) depending on the stimulus^7,8^. Pro-inflammatory M1 secretes IL-6 and IL-1β and mediates inflammatory response, while anti-inflammatory M2 secretes IL-10 and other anti-inflammatory factors and performs tissue repair function^9^. In obesity and insulin resistance, macrophages tend to exhibit M1 polarization, that is, the M1/M2 ratio tends to increase; the levels of pro-inflammatory factors increase, while anti-inflammatory factors decrease; M1 polarization not only lead to the decrease of blood glucose utilization and repair of inflammatory damage in visceral tissues, but also lead to the impairment of islet β cell function and even apoptosis^10^. Emerging evidence has suggested that macrophages play a critical role in insulitis^11^. It is worth noting that M1 polarization drives pathogenesis and progression of T1DM by exacerbation of inflammatory responses via inflammatory cytokines^12,13^. For example, one study stated that M1 macrophages promoted destruction of β cells in T1DM mice through excessive production of IL-1β^14^.

Most studies in this area have focused on the changes of CD4^+^ T cells and macrophages in the pancreas^15–18^, but T1DM can cause multiple-organ damage and serious complications. In this study, we explored the changes of immune cells in diabetic mice. We described the changes of CD4^+^ T cells and macrophages in spleen, pancreas and kidney of streptozocin-induced T1DM mouse model. Our work could help to have a comprehensive understanding on the changes of CD4^+^ T cells and macrophages during an inflammatory process of T1DM, which could provide an experimental basis for the subsequent research of T1DM-related medication, especially about the CD4^+^ T cells and macrophages regulation.

## Materials and methods

### T1DM modeling

All procedures and experiments were performed in accordance with all relevant guidelines and regulations including a protocol approved by the committee of Experimental Animal Ethics of Jilin University, Changchun, China (permit number: SYXK 2018-0001) and in accordance with the ARRIVE guidelines (https://www.arriveguidelines.org). We used Two groups of Three male BALB/C mice (6–8 weeks old) and bred them under 50–60% humidity and 22–25 °C temperature in a 12/12-h light dark cycle in the Animal Center of the School of Basic Medical Sciences, Jilin University, China.

The model of T1DM was established by intraperitoneal injection of streptozocin (60 mg/kg) for 5 days. After 1 week, the blood glucose of the model mice was measured. The diagnostic standard of DM was that the level of blood glucose of three consecutive times was equal to or higher than 16.7 mmol/l.8 weeks after the diagnosis of DM, all animals were sacrificed by cervical dislocation for further study. The major internal organs (the spleen, kidneys, pancreas) were harvested for Western blot (stored at − 80 °C until extraction), histological analysis (fixed in 10% formalin solution, preserved in wax blocks) and Flow cytometric analysis (tissue cells were isolated, and a single-cell suspension was made for the experiment).

### Western blot

Kidney tissue (from − 80 °C freezer, about 100 mg), was placed in 1 ml of RIPA (Beyotime) and 10ul of PMSF (Beyotime) to break down and extract protein. The concentration of renal tissue protein in different groups of mice was detected by BCA kit. The 40ug protein sample was placed in 15% SDS-PAGE gel for electrophoresis (80 V, 30 min and then 120 V, 90 min), transferred to PVDF membrane (1 h), sealed with 5% skimmed milk powder (1 h), and then incubated overnight with rabbit anti-mouse IL-6 antibody (1:2000, Genetex), rabbit anti-mouse IL-1 β antibody (1:2000, Abcolonal) and rabbit anti-mouse Il-10 antibody (1:2000, Genetex) in 4 °C refrigerator. The next day, they were incubated with goat anti-rabbit antibodies (1:5000, Proteintech) for 1 h and observed protein bands with ECL kit.

### Immunohistochemical staining

For staining of tissue sections, slides were deparaffinized in xylene and rehydrated in graded alcohol solutions. Sodium citrate was restored under high pressure, using endogenous peroxidase blockers and non-specific dye blockers (MXB Biotechnologies). Sections were incubated overnight at 4 °C with 1:200 diluted rabbit anti mouse F4/80 antibody (Affinity) and 1:100 diluted rabbit anti mouse CD4 antibody (Abcolonal). The next day, relevant goat anti-rabbit IgG secondary antibody (MXB Biotechnologies) drops were incubated, rinsed with PBS, DAB re-stained, and hematoxylin cell nuclei re-stained. The sections were dehydrated in a concentration gradient of ethanol and xylene, sealed with neutral resin, and then observed under the microscope.

### Flow cytometry

Spleen tissues were ground and separated into suspensions; the suspensions were centrifuged at 1500 rpm for 5 min; the supernatant was discarded; red blood cell lysis buffer (Biyuntian Company) was added for 5 min and then filtered through a 200-mesh screen, centrifuged at 1500 rpm for 5 min; the supernatant was discarded; the precipitate was resuspended in PBS. The phenotypes of these cells were analyzed by Flow cytometry. Before flow cytometry, 20 μl of cell suspension was diluted with 20 μl of trypan blue and the numbers of live (trypan blue negative) and dead cells (trypan blue positive) were counted under a microscope. Cell surface staining: anti-CD4-FITC(Biolegend), anti-CD4-PE(Biolegend), anti-CD25-FITC (Biolegend), APC-labeled anti-lineages cocktail including Ly6G, CD3, and B220(Elabscience), anti-CD11b-PE (Biolegend), anti-CD115-PE-Cy5.5(Biolegend) were added to the corresponding samples. Intracellular staining: the cells were fixed and permeabilized with Fixation/Permeabilization Concentrate and Fixation/Permeabilization Diluent (1:3) (Biolegend), Permeabilization Buffer (Biolegend), and then add anti-IFN-γ-PE (Biolegend), anti- IL-4-APC (Biolegend), anti-IL-17-PE (Biolegend), anti-FOXP3- PerCP-Cy5.5 (Biolegend) to the corresponding samples.

Kidney tissue was cut up and digested with 0.2% Collagenase IV (37 °C incubator for 1 h), the product was centrifuged at 1500 rpm for 10 min and the supernatant was discarded, red blood cell lysis buffer (Biyuntian Company) was added for 5 min and then filtered through a 200-mesh screen, 1500 rpm for 5 min and the supernatant was discarded, the precipitate resuspended in PBS and analyzed for phenotypes by flow cytometry. Before flow cytometry, 20 μl of cell suspension was diluted with 20 μl of trypan blue and the numbers of live (trypan blue negative) and dead cells (trypan blue positive) were counted under a microscope. Cell surface staining: anti-F4/80-APC (Biolegend), anti-CD11b-PE (Biolegend) and CD86-FITC (Biolegend) were added to the corresponding samples. Intracellular staining: to detect intracellular CD206, the cells were fixed and permeabilized with Fixation Buffer (Biolegend) and Intracellular Staining Permeabilization Wash Buffer (Biolegend), and then stained with anti-CD206-PE-Cy7(Biolegend).

### Statistical analysis

Unpaired t test was used for comparison between the two groups and two-way ANOVA tests with Sidak's post hoc analyses were used for multiple comparisons. Differences were significant if *P* < 0.05. GraphPad Prism 8.3.0 was used for all statistical analysis.

### Ethics approval and consent to participate (Human Ethics, Animal Ethics or Plant Ethics)

The animal study was reviewed and approved by the Experimental Animal Center of Jilin University, Changchun, China (permit number: SYXK 2018-0001).

## Results

### T1DM model induced by streptozocin

**Figure 1:**
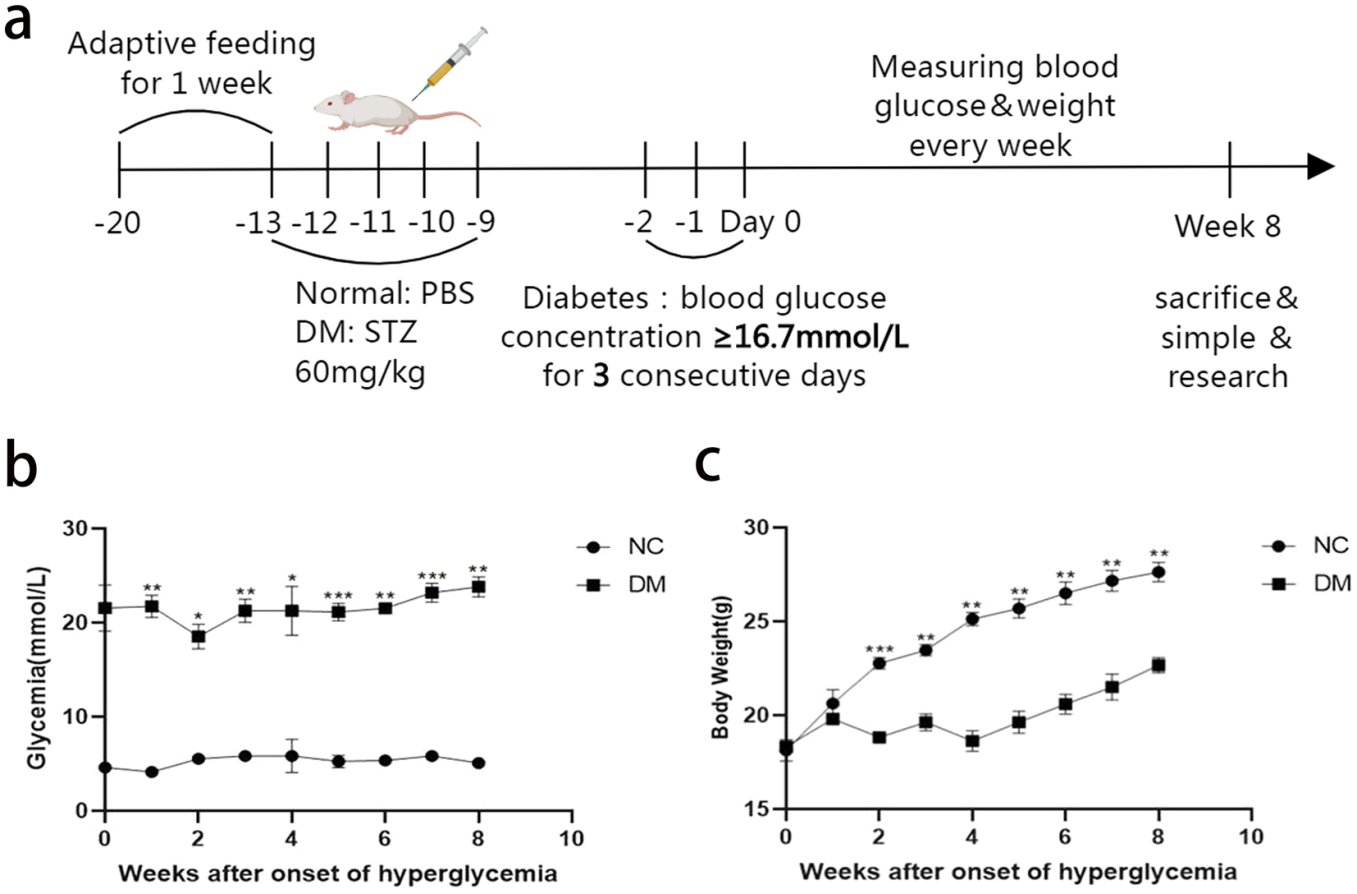
Flow diagram of experiments and conventional indicators of mice. Timetable and flowchart of mice treatment and identification of mice DN model (**a**). Fasting blood glucose was measured every 7 days in normal and DM mice (**b**), the changes in body weight of normal and DM mice were measured every 7 days (**c**). NS showed no significant difference (*P* > 0.05), * *P* < 0.05, ** *P* < 0.01, *** *P* < 0.001. NC, normal control, DM, diabetes mellitus.

Streptozocin was used to establish a mouse model of T1DM. All experimental animals were sacrificed after 8 weeks of T1DM. At the same time, tissue samples are kept for further study (Fig. 1a). To observe the changes of blood glucose in mice, fasting blood glucose was measured every 7 days. The fasting blood glucose in normal mice fluctuated within the normal level (3.5–6.9 mmol/l), while the blood glucose level of the DM group was higher than 16.7 mmol/l (Fig. 1b). At the same time, the weight changes of mice were monitored during the feeding process. As shown in Fig. 1c, after the T1DM mouse model was established, there was no significant weight gain in the DM group from week 1 to week 4 compared with the normal group. Although weight gain began at week 5, weight gain in the DM group was significantly lower than that in the normal group.

### The change of the proportion of CD4^+^ T cells and its subtypes in spleen, pancreas and kidney

**Figure 2:**
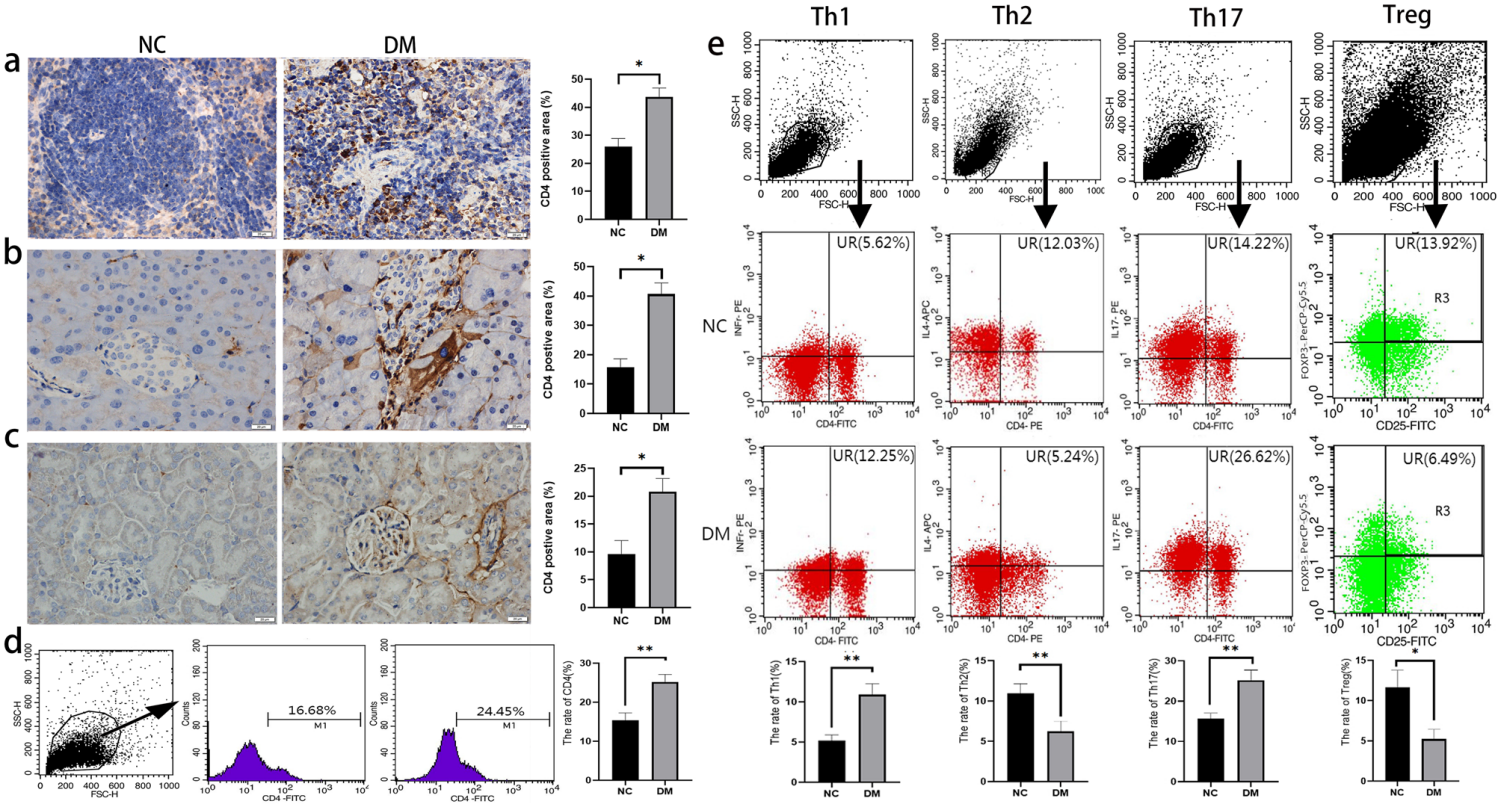
The change of the CD4^+^ T cells and its subtypes in different organs. CD4-positive cells were detected by Immunohistochemical staining in different organs and their positive area was evaluated, spleen (**a**), pancreas (**b**), kidney (**c**). Changes in the proportion of CD4^+^ T cells (CD4^+^) in the spleen of each group was determined by Flow cytometry (**d**). Changes in the proportion of CD4^+^ T cells’ subtypes in the spleen of each group was determined by Flow cytometry. Th1 cells: CD4^+^ and IFN-γ^+^; Th2 cells: CD4^+^ and IL-4^+^; Th17 cells: CD4^+^ and IL-17^+^; Treg cells: CD4^+^, CD25^+^ and Foxp3^+^ (**e**). Scale Bar = 20 μm, * *P* < 0.05, ** *P* < 0.01. NC, normal control; DM, diabetes mellitus.

In order to study the infiltration of T cells in spleen, pancreas and kidney, CD4 (T cell marker) immunohistochemical staining were performed on these organs. The results showed that the ratio of CD4^+^ cells in spleen, pancreas and kidney of normal mice was low, while the ratio of CD4^+^ cells in the above organs of DM mice was significantly increased (*P* < 0.05), among which the increase of CD4^+^ cells in spleen and pancreas was more obvious (Fig. 2a–c). Spleen is an important peripheral immune organ, where most of the immune cells proliferate. And the change of the proportion of immune cells in the spleen can reflect the immune state of DM mice to some extent^19,20^, so we used flow cytometry to measure the ratio of CD4^+^ T cells and their subtypes in normal and DM groups. Compared with normal mice, the proportion of pro-inflammatory CD4^+^ T subtypes, Th1 cells and Th17 cells in DM group was significantly increased, and the proportion of Tregs cells and Th2 cells was significantly decreased (Fig. 2d, e).

### The change of monocytes and macrophages in spleen, pancreas and kidney

**Figure 3:**
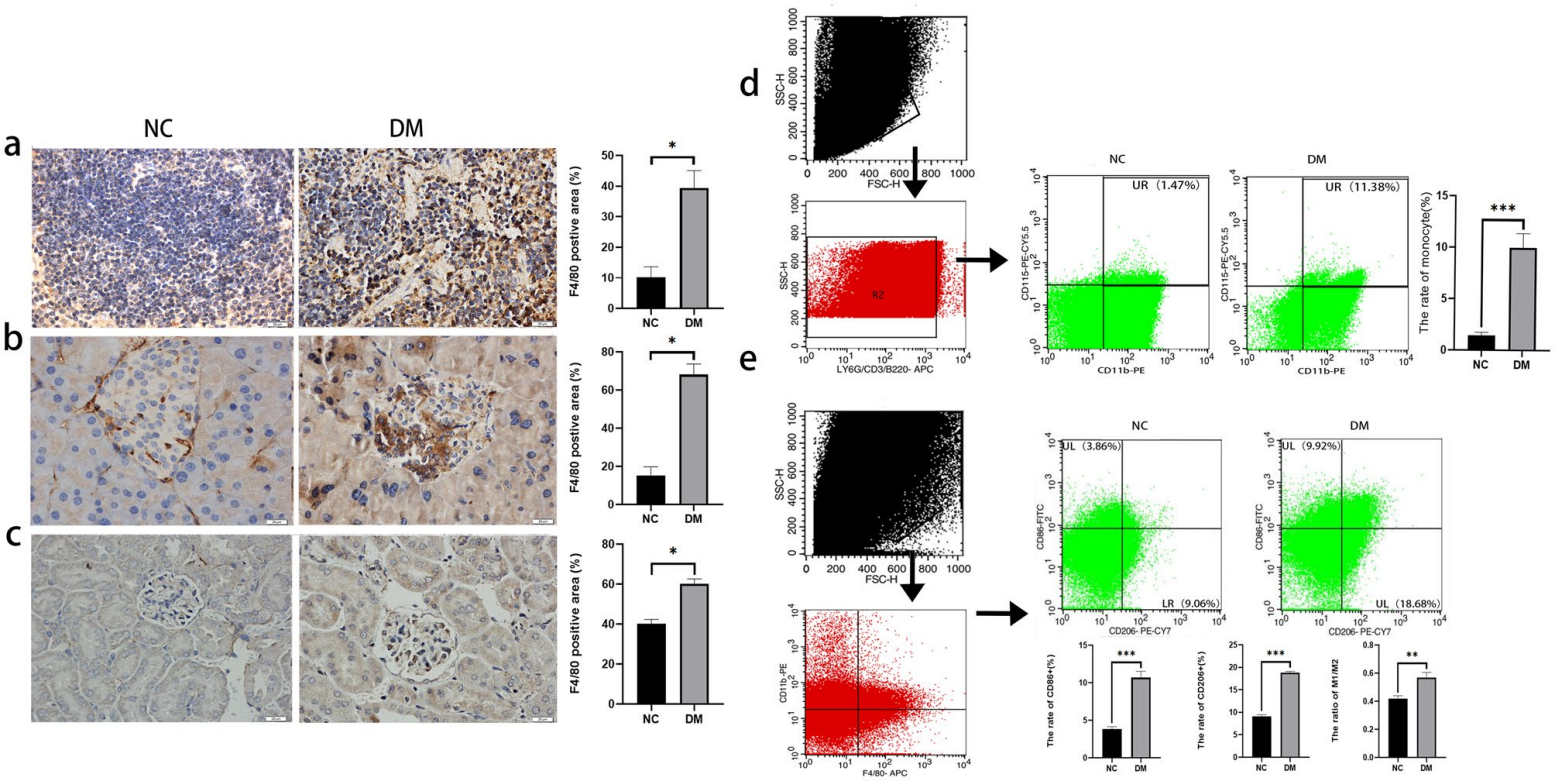
The change of the macrophages in different organs. F4/80-positive cells were detected by Immunohistochemical staining in different organs and their positive area was evaluated, spleen (**a**), pancreas (**b**), kidney (**c**). Changes in the proportion of monocytes (Ly6G^–^, CD3^–^, B220^–^ and CD11b^+^, CD115^+^) in the spleen of each group was determined by Flow cytometry (**d**). Changes in the proportion of macrophage (CD11b^+^ and F4/80^+^) and its subtypes (M1(CD86^+^) and M2(CD206^+^)) in the kidney of each group was determined by Flow cytometry (**e**). Scale Bar = 20 μm, * *P* < 0.05, ** *P* < 0.01, *** *P* < 0.001. NC, normal control; DM, diabetes mellitus.

In order to study the infiltration of macrophages, F4/80(macrophage marker) immunohistochemical staining were performed in spleen, pancreas and kidney. The results showed that the proportion of F4/80^+^ macrophages in spleen, pancreas and kidney of normal mice was lower, while the proportion of F4/80^+^ macrophages in the above organs of DM mice was significantly increased (*P* < 0.05), and the increase of macrophages in spleen and pancreas was also more obvious (Fig. 3a–c). As it was mentioned previously, the spleen is an important peripheral immune organ in the body and the main storage organ for immune cells includes monocytes, and the change of the proportion of monocytes in the spleen can reflect systemic inflammatory state. As for monocytes (Fig. 3d), the proportion of monocytes in the spleen of the DM group was significantly higher than that of the normal group. DM can lead to multiple target organ damage and serious complications. Oxidative stress, hyperglycemia and hyperlipidemia can promote multiple microvascular complications during the development of DM, of which renal injury is one of the most serious microvascular complications. Diabetic nephropathy is considered to be an inflammatory disease, and macrophages play an important role in the development of inflammation^21^, so we measured the proportion of macrophages subtypes in the kidneys of different groups of mice. The proportion of both M1(CD86^+^) and M2(CD206^+^) was increased in the kidneys of mice in the DM group relative to the normal group, and we noticed that the ratio of M1/M2 in the DM group also increased compared with the normal group (Fig. 3e).

### The expression of renal inflammatory factors was increased in the course of T1DM mice

**Figure 4:**
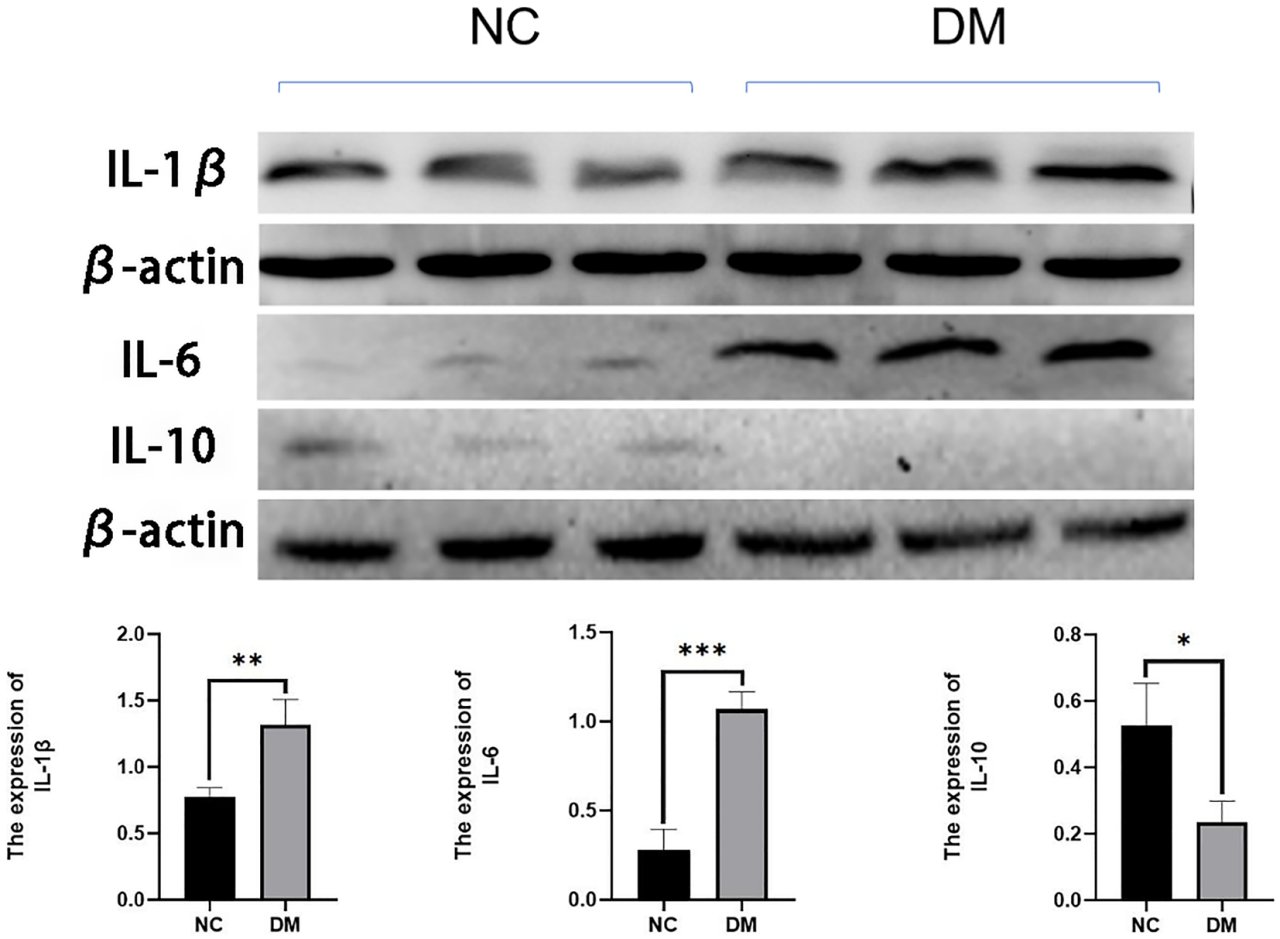
The representative pictures and Quantitative analysis of IL-6, IL-1 β and IL-10 in kidney tissue of mice in each group. The gels/blots were cropped from different parts of the different gels and grouped. The original blots/gels are presented in Supplementary Fig. 1. * *P* < 0.05, ** *P* < 0.01, *** *P* < 0.001. NC, normal control; DM, diabetes mellitus.

T1DM is known to promote a variety of microvascular complications, among which kidney injury is one of the most serious microvascular complications. According to immunohistochemical results, in the course of T1DM, T cells and macrophages can infiltrate kidney tissue, promoting local inflammatory response, so we analyzed the expression of pro-inflammatory IL-6, IL-1β and anti-inflammatory IL-10 in the kidney. The expression of IL-6 and IL-1β in kidneys of the DM group was significantly higher than that of the normal group, and the increase of IL-6 expression was more significant compared with IL-1β, while the expression of IL-10 in the normal group was slightly higher than that in DM Group (Fig. 4). It indicated that the process of diabetic kidney injury was accompanied by a local inflammatory response in the kidney.

## Discussion

DM is a metabolic disease with inflammatory response. The systemic and local inflammatory responses have an important role in the development and progression of DM, in which CD4^+^ T cells and macrophages are closely associated with T1DM^22–24^. During the DM, these two types of immune cells infiltrate the major organs, triggering an inflammatory response. Multi-streptozocin protocol is nowadays a routine method used in type 1 diabetes model building. In contrast to single high-dose streptozocin, which causes massive necrosis of the pancreaticβ-cell mass and has potential toxicity to other organs, multiple low doses of streptozocin initiate an insulitis similar to that observed in T1DM and moderate hyperglycemia^25^. In this experiment, we established a multi-streptozocin-induced T1DM model, and compared the CD4^+^ T cells and its subtypes, infiltration of CD4^+^ T cells and macrophages in different organs, cell proportion changes, and so on. Furthermore, the changes of some immune cells in T1DM inflammation were preliminarily studied. During the development of T1DM, many organs were infiltrated by immune cells, and CD4+ T cells promote the activation and infiltration of macrophages aggravating the inflammatory state^26–28^, so the spleen, pancreas and kidney of mice were stained by immunohistochemistry, and the degree of infiltration of CD4^+^ T cells and macrophages was observed in the DM Group and normal group.

CD4^+^ T cells play an important role in the occurrence and development of T1DM, and can mediate the destruction of pancreatic β cells, thus inducing the occurrence of type 1 diabetes^29,30^. The immunohistochemical results of mouse suggest that, compared with the normal group, the proportion of CD4^+^ T cells and macrophages in the pancreas of DM mice were significantly increased. The literature reports that there was acute infiltration of CD4^+^ T cells in the islet of T1DM patients, the pro-inflammatory cytokine secreted by CD4^+^ T cells promoted the apoptosis of islet β cells and it has been found that when T cells from T1DM mice are transplanted into normal mice, the islet β cells of normal mice are destroyed and then diabetes occurs^31^. In our research, the change of CD4^+^ T cells positively correlates with macrophages in pancreas, these results could suggest the relationship between CD4+ T cells and macrophages during the progress of T1DM. As an important peripheral immune organ, most immune cells proliferate in the spleen, and the change in the proportion of immune cells in the spleen can reflect the inflammatory reaction of DM mice to some extent^19,20^. According to the results, the proportion of CD4^+^ T cells and macrophages in the DM group was higher than that in the normal group, which was related to the increased mobilization of immune cells in the spleen during the inflammatory reaction. It has been reported in the literature that diabetic renal injury is associated with marked infiltration of immune cells^32^. The immunohistochemical results of mouse kidneys suggest that, compared with the normal group, the proportion of CD4^+^ T cells and macrophages in the kidney of DM mice were significantly increased, and there was infiltration in both glomerulus and renal tubules, which suggested that immune cells might increase the local inflammatory reaction in both glomerulus and renal tubules.

Notably, the proportion of CD4^+^ T cells and macrophages in the spleen, pancreas and kidney of DM mice was higher than that of normal mice, especially in the pancreas. This may be related to the early injury of pancreas and persistent inflammatory cell injury in DM mice. The above results show that the change of CD4^+^ T cells positively correlate with macrophages in these organs during inflammatory response of T1DM. It is suggested that there may be a synergistic relationship between CD4^+^ T cells and macrophages in the progress of T1DM.

Immunohistochemical staining of the spleen revealed infiltration of CD4^+^ T cells, so we used Flow cytometry to determine the proportion of CD4^+^ T cells and their subtypes. Compared with normal mice, the proportion of CD4^+^ T cells, Th1 cells and Th17 cells in DM Group increased significantly, while the proportion of Tregs cells and Th2 cells decreased significantly. CD4^+^ T cells can differentiate into different subtypes, among which helper T cells (Th), Th1 cells and Th17 cells are pro-inflammatory cells, while Th2 cells and regulatory T cells (Tregs) are anti-inflammatory cells^33–35^. CD4^+^ T cell subtypes and their secreted cytokines play an important role in T 1 DM^23,36,37^. The ratio of Th1 and Th17 cells in T1DM patients increases, and the expression of pro-inflammatory factors such as IL-17A, IFN-γ, and IL-2 secreted by them is up-regulated, which aggravates the inflammatory response in the body, and the ratio and function of Tregs cells are weakened, and the expression of anti-inflammatory factors such as IL-10 and TGF-β is down-regulated, leading to a decrease in the body's ability to suppress inflammation^38–40^. It has been reported that Mesenchymal stem cells have been shown to have an immunosuppressive effect by binding PD-L1 to PD-1 receptors on the surface of CD4^+^ T cells for the treatment of diabetes. At the same time, MSCs can effectively reduce the insulin requirement and blood glucose level of diabetic patients^41^. Previous report has pointed out that overexpression of PD-L1 can induce islet regeneration, restore insulin secretion and maintain normal blood glucose, and inhibit the proliferation of infiltrating CD4^+^ T cells^42^. The expression of PD-L1 was increased in T1DM mice treated with MSCs, and the secretion of anti-inflammatory factors by Tregs and Th2 cells was increased, which induced the immune response to change to Th2-like, thus alleviating the injury caused by diabetes^43,44^. Therefore, the treatment of T1DM by regulating PD-L1/PD-1 pathway and CD4^+^ T cells may be a very promising method.

The spleen is an important peripheral immune organ in the body and the main storage organ for monocytes. When inflammation occurs, monocytes can leave the spleen and go to the site of inflammatory injury^45^. Therefore, the proportion of monocytes in spleen can indirectly reflect systemic inflammatory state.

DM can cause multiple target organ damage and serious complications, among which renal injury is one of the most serious microvascular complications. Diabetic kidney injury was once thought to be associated primarily with metabolic and Hemodynamics changes, but in recent years, there has been increasing evidence that inflammation plays an important role in the progression of diabetic kidney injury^46,47^. Throughout the course of diabetes, inflammation in the kidneys determines the degree of progression of kidney damage, and diabetic nephropathy is associated with systemic and local inflammation in the kidney. Renal inflammation is characteristic of diabetic nephropathy and is closely associated with disease progression^48^. Inflammatory response is a risk factor for glomerulosclerosis and tubulointerstitial fibrosis by inducing extracellular matrix deposition and proliferation and differentiation of myofibroblasts. Thus, blocking renal inflammation may be a strategy for treating diabetic nephropathy^49^. The local infiltration of inflammatory cells in kidney is related to many inflammatory factors, such as IL-6, IL-1β and so on. Compared with normal group, the expression of IL-6 and IL-1β in kidneys of DM Group is obviously higher, with the most pronounced change in IL-6 expression, while the expression of IL-10 in the normal group was slightly higher than that in the DM group. According to the literature, macrophages can be classified into classically activated M1 type and substitutionally activated M2 type^7,8^. In the process of inflammation, M1 macrophages secrete IL-6 and IL-1β^50^. M2 macrophages secrete IL-10 and produce an anti-inflammatory microenvironment^51^. Therefore, we measured the ratio of M1/M2 macrophages in mouse kidneys using the Flow cytometry method. The results showed that the ratio of M1/M2 in the kidney of DM Group was higher than that of normal group, which was different from the previous report^52^, but the ratio of M1/M2 in mice in the DM group was higher than that in the normal group, which may be related to the fact that the mice in this experiment were kept for a shorter period and were still in the early stage of local inflammation in the kidney, and M1 macrophages have a higher polarizing rate than M2. In future experiments, we may be able to measure the ratio of M1/M2 in mouse kidneys at different time points in order to dynamically monitor the changes.

## Conclusion

Our study suggests that CD4^+^ T cells and macrophages increase, while the inflammatory immune response system is activated in the development of T1DM. CD4^+^ T cells positively correlated with macrophages in the pancreas and kidney of T1DM. CD4^+^ T cells turn to pro-inflammatory subtypes in the spleen of T1DM, while macrophages turn to pro-inflammatory subtypes in the kidney of T1DM. Therefore, regulation of CD4^+^ T cells and macrophages may be a potential target for T1DM and kidney complications.

## Supplementary Information

Supplementary Figure 1.

## Data Availability

The data and materials used and/or analyzed during the current study are available from the corresponding author on reasonable request.
