# Supplementary figures and images for: Changes of macrophage and CD4+ T cell in inflammatory response in type 1 diabetic mice
Source: Sci Rep. 2022 Sep 2;12:14929. doi: 10.1038/s41598-022-19031-9 (PMC9440103; doi:10.1038/s41598-022-19031-9)

**
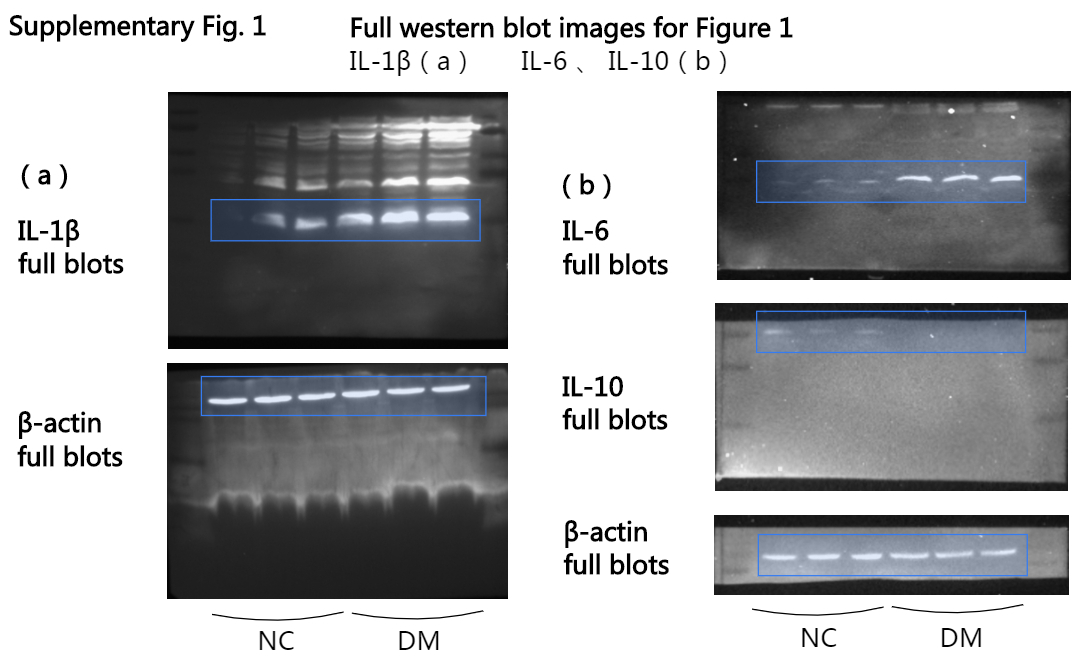
**

**Supplementary Figure 1** Full western blot images for Figure 1: （**a**）IL-1β,（**b**）IL-6 , IL-10.

Supplement: Supplementary file 1 — Supplementary Figure 1. [file 41598_2022_19031_MOESM1_ESM.docx]
